# Bad Smells

**Published:** 1871-07

**Authors:** 


### BAD SMELLS

Do not cause disease. Slaughter-houses look very uninviting, and spread very disagreeable odors in every direction. Not less distasteful are the emanations from various manufactories. The dead bodies of birds and beasts and men cause a revolting noisomeness, but none of these originate any specific diseases. Many years ago it became necessary in Paris to remove the corpses from a graveyard; in some localities the stench was so insufferable, that if the men remained at work longer than a few minutes at a time, they fairly fainted away; so they took turns at short intervals, with the result, that the work was accomplished and not a single laborer was even taken sick. There is a wisdom in ordaining that the bodies of the dead should send out insufferable odors, for it compels their sepulture; otherwise, affection would retain the forms of the loved and lost in their dwellings for an indefinite time.

It is not intended to say that bad smells never kill, for they may be so concentrated that the air may not have nourishment enough to sustain life; for example, if common illuminating gas escapes into a close room where a man is sleeping, he will die in a few hours; the idea sought to be conveyed is, that bad smells, under the ordinary circumstances of spreading in the air, especially those arising from the dead animal substances, and from many other sources, do not cause disease.

But the element of half the diseases of mankind, of all the plagues and pestilences which have, in the season of a few short weeks, swept countless thousands into their graves, which have at times almost depopulated whole villages and towns and cities in a month, is the result of decaying vegetation, not of decaying animal matter, and has no smell at all; it is in the air, but neither sight, nor sense, nor taste can detect it; but it is there, and in a night has before now killed half a community; it is the "miasm," the emanation rising from the ground where leaves and weeds and wood are in process of decomposition, by the joint action of heat and moisture; hence pestilence and plague reign rampant in warm weather, — in warm localities, never in cold. Pestilence and plague never swept across a desert, because there was no vegetation there. The practical use to be made of these facts is simply to prevent persons bestowing all their efforts, in times of great sickness, to the suppression of bad smells, for it may be time and labor lost, while the death-dealing element still slays its thousands. A dozen slaughter-houses together, on the side of a mill-pond, sending their stench for miles around, might be leveled to the ground, and still disease and death would spread, as long as the mill-pond was covered with leaves and rotten wood and weeds; they may give out no appreciable odor, but the emanations arising from them are most destructive of health.

